# The younger women’s wellness after cancer program: results from feasibility testing in Aotearoa New Zealand (the ‘Kōwhai study’)

**DOI:** 10.1007/s00520-025-09601-8

**Published:** 2025-06-05

**Authors:** Janine P. PORTER-STEELE, Katrina J. SHARPLES, Bobbi B. LAING, Sarah BENGE, Sarah M. BALAAM, Natalie K. VEAR, Michael P. N. FINDLAY, Ian D. CAMPBELL, Marion J. J. KUPER-HOMMEL, Debra J. ANDERSON, David J. PORTER, Alexandra L. MCCARTHY

**Affiliations:** 1 Griffith Health Group Executive, Griffith University, Gold Coast Campus, Southport, Australia; 2 School of Nursing, Midwifery and Social Work, The University of Queensland, Level 3, Chamberlain Building (35), St Lucia, Brisbane, QLD 4072 Australia; 3 Wesley Choices Cancer Support Centre, The Wesley Hospital, Auchenflower, Australia; 4 Cancer Trials New Zealand, The University of Auckland, Auckland, Aotearoa New Zealand; 5 University of Otago, Dunedin, Aotearoa New Zealand; 6 School of Nursing, University of Auckland, Auckland, Aotearoa New Zealand; 7 Department of Surgery, Waikato Hospital, Hamilton, Aotearoa New Zealand; 8 Department of Oncology, Waikato Hospital, Hamilton, Aotearoa New Zealand; 9 University of Technology Sydney, Ultimo, Australia; 10 Department of Oncology, Auckland Hospital, Auckland, Aotearoa New Zealand; 11 Mater Research Institute, South Brisbane, Australia; 12 Wesley Research Institute, The Wesley Hospital, Auchenflower, Australia

**Keywords:** Oncology, Breast cancer, Physical activity, Exercise, Diet

## Abstract

**Purpose:**

This paper reports the feasibility testing of the Younger Women’s Wellness after Cancer Program in Aotearoa New Zealand (the ‘Kōwhai Study’) by examining (a) intervention uptake, adherence, and sustainability over time and (b) the feasibility of the proposed trial methods.

**Methods:**

Participants were female, aged between 18 and 50 years, and had completed stage I or II breast cancer treatment within the previous 24 months. They also had internet access. Participants were randomly allocated 1:1 to the intervention or control group. The intervention consisted of systematic 12-week internet-based individualised coaching based on promoting a sustainable healthy lifestyle and managing treatment side effects. The control group received the usual care expected from their treating healthcare teams. Outcomes were measured at baseline, week 12, and week 24. The sample size for the study was 60 participants.

**Results:**

Target *N* = 60 was reached, with groups evenly matched socio-demographically. All participants completed all questionnaires at baseline (average 15–20 min to complete). Complete intervention/control questionnaire data was provided at week 12 by 76.7%/73.3% and week 24 by 86.7%/70.0% respectively. The 12-week intervention was completed by 28/30 participants. Free-text data strongly indicated intervention and study processes were acceptable and feasible to participants.

**Conclusion:**

In Aotearoa New Zealand, the intervention is valued by the target group and the study processes are feasible.

Trial registration

Australian New Zealand Clinical Trials Registry, ACTRN12620000260921. Registered on 27 February 2020.

**Supplementary Information:**

The online version contains supplementary material available at 10.1007/s00520-025-09601-8.

## Introduction

Younger women (< 50 years) treated for breast cancer represent a growing population in Aotearoa New Zealand (NZ) [1]. Whilst their treatment outcomes have improved, they face a considerable risk of treatment-related chronic conditions such as psychological distress, diabetes, and obesity [2, 3]. This poses a significant public health issue [4]. The Younger Women’s Wellness after Cancer Program (YWWACP), developed in Australia, is a whole-of-lifestyle intervention that promotes physical activity, optimal diet, smoking cessation, alcohol reduction, plus strategies for sleep, stress, sexual wellbeing, and treatment-related menopausal symptom management [5, 6]. No similar holistic program is available in Aotearoa NZ. Our plan was to test whether the Australian-developed YWWACP could improve the quality of life and health of younger women with breast cancer in Aotearoa NZ. Before proceeding with large scale national testing, we conducted this feasibility study (the Kōwhai Study, *N* = 60) to refine the trial methodology and address issues encountered in the Australian pilot.

Approximately 30% of all breast cancers occur in younger women. Roughly 4000 younger women (aged ≤ 50 years) are diagnosed with breast cancer in Aotearoa NZ each year, with incidence rising 18% in the last decade [7]. Advances in treatment have improved survival, with 87% alive at 5 years [8]. However, many women experience significant treatment-related health problems including osteoporosis (HR 1.27 95%CI, 1.17–1.39) and diabetes (HR 1.24, 95%CI, 1.10–1.41) [9]. Obesity, neuropathy, lymphoedema, disturbed body image, fear of recurrence, and sleep disorders are also common [10]. Younger women face additional treatment-induced physiological alterations such as ovarian failure, early and severe menopausal symptoms, joint pain, genitourinary dysfunction, and compromised bone health [11]. They report poorer emotional wellbeing, impaired sexual health, higher rates of clinical depression, and amplified psychological distress [3]. This leads them to rate their health as ‘poor’ or ‘fair’ compared to matched controls [12].

Whilst many of these issues can be prevented or mitigated by consistent engagement in health-promoting activities after treatment, many women lack guidance on managing their health [13]. They are more likely to die of modifiable treatment effects than of their primary cancer [14]. Given higher survival rates post-treatment, wellbeing is recognised globally as a significant public health issue, from which Aotearoa NZ is not exempt. Targeted intervention, which is currently scarce for this group, is needed.

The Australian-developed YWWACP intervention aims to address these challenges through a flexible tailored approach promoting self-management of chronic disease risk irrespective of geographic location. The YWWACP is delivered virtually, leveraging internet access (~ 90% of New Zealanders had access in 2018) [15], offering a low-cost accessible support option nationwide. Its precursor has previously been trialled in women of any age following breast cancer in Australia (*N* = 351), showing improvements in physical activity, health-related quality of life, self-efficacy (exercise and diet), sleep, and menopausal symptoms [16]. The program was adapted to the YWWACP for further pilot testing in younger Australian women (average age = 38 years; *N* = 41) (*data not published*). It achieved 66% retention at week 12 with a significant increase in physical activity (*p* = 0.017) (*data not published*). When interviewed at week 12, those who withdrew from the study often cited measurement burden as a critical factor in their decision. Based on this feedback, we have changed the nature and length of the assessments for this Aotearoa NZ study.

Australian development work [17] identified two critical methodological issues requiring resolution before large-scale trialling in Aotearoa NZ. Firstly, whilst retention was strong, recruitment was unrepresentative, with all participants being Australian European, generally affluent and well-educated. Socially and demographically disadvantaged women face higher cancer incidence and poorer outcomes. Therefore, we needed to assess uptake, accessibility, and acceptability in a diverse group of young NZ women with breast cancer including those who identify as Māori (17%), Pacific Islander (7%), or Asian (9%), or who reside in areas of deprivation. A separate feasibility study specifically for Māori women (Māreikura Tū Kōwhai Māori) has also been trialled and will be reported separately (*paper forthcoming*). Secondly, during preliminary work [17], measurement burden was substantial and repetitive, with assessments reportedly taking up to 90 min at each of three measurement time-points. For this feasibility study in Aotearoa NZ, we streamlined assessments whilst maintaining essential intervention impact measures. The aims of this feasibility study are to determine the potential (1) to translate the YWWACP intervention to a broader population base, and (2) for success of the main trial internationally [18].

## Methodology

### Participants

Eligible participants were aged 18–50 years; had completed any intensive treatment (surgery, chemotherapy, radiotherapy) for stage I–II breast cancer in the previous 24 months (were at least 4-week post-intensive treatment) but could be on endocrine therapy; were NZ residents; had internet access; and could speak and read English. Exclusions included a current diagnosis of metastatic or advanced cancer, and any clinical contraindication precluding safe participation.

### Recruitment strategy

Recruitment was conducted through social media pushes via community support group research partners (Cancer Society Auckland Northland Division, Breast Cancer Foundation, Breast Cancer Coalition Aotearoa, and Pinc and Steel). Study fliers were also distributed at presentations to engage clinicians and participants at Auckland and Waikato Hospitals. Women registered for the study using a dedicated web address, allowing them to review the study material privately before completing online consent forms. Consenting women were allocated a unique ID number and registered on the trial database. Any reasons given for non-participation were recorded.

### Randomisation

Participants were randomly allocated 1:1 to the intervention or control after baseline data collection. Randomisation was carried out using the standard Cancer Trials NZ (CTNZ) web-based randomisation process using blocking over time, with random block sizes to aid concealment of allocation. Randomisation was performed by a member of the research team who was independent of recruitment and data collection.

### Intervention

The YWWACP is grounded in Bandura’s Social Cognitive Theory [19], highlighting the dynamic interaction between personal factors, behaviours and the environment, alongside individuals knowledge and skills in shaping self-efficacy for health behaviour change. It aimed to empower women to make gradual, achievable adjustments to their lifestyle, strengthening self-efficacy and fostering long-term healthy habits. The intervention incorporated international recommendations for physical activity, diet, alcohol moderation, smoking cessation, alongside strategies for sleep, stress, menopausal symptoms, and sexual health over 12 weeks [6]. Personalised support was provided through three coaching sessions with a trained cancer nurse specialist to meet participants’ individual goals and functional capacities. No components of the program book [6] were adapted prior to testing in the main Kōwhai study, but were adapted for the study in Māori (*not reported here*).

Key evidence-based exercise strategies included goal setting and self-monitoring, exercising with a friend, tracking step counts, and maintaining exercise logs [20, 21]. If deemed required by the intervention nurse specialist, a participant would be referred to an exercise physiologist to enable further tailoring of exercise activities to individual fitness levels and support long-term adherence. Dietary improvements were encouraged through explanation of positive nutritional choices to reduce chronic disease risk, with personalised nutritional coaching focusing on individual preferences to encourage greater adherence [22, 23]. Motivational interviewing techniques were employed to enhance motivation and self-efficacy to reduce alcohol intake [24]. Smoking and vaping cessation support was achieved by referral to a general practitioner in connection with QUITLINE [25]. Sleep concerns were explored and discussed in the nurse specialist consultations and participants were encouraged to use the ‘BETTER’ (Sleep) acronym to promote sleep habits. Additionally, relaxation and mindfulness techniques were used to improve stress resilience and overall wellbeing [26]. For menopausal symptoms, strategies ranged from simple lifestyle tips for managing hot flushes, cognitive function, joint pain, and irritability, to more advanced options, including medication, depending on the severity of symptoms and participant preferences [6]. Finally, strategies to support sexual health included pelvic floor exercises, which strengthen muscles to improve sexual function and reduce incontinence [27, 28]. Supplementary tables 1–3 provide further detail on content, delivery mode, and timing of the intervention.

### Standard care

Participants allocated to the standard care group received general information at the discretion of their usual healthcare professionals during clinic visits about the management of all symptoms. This included the general information and advice available to them from their treating team about physical activity, diet, tobacco, and alcohol abstinence, plus information about support services such as Pinc and Steel [29] and the Aotearoa NZ Divisions of the Cancer Society [30]. Participants’ use of these services, and any interventions they offer, were documented. Control participants were offered a hard copy of the YWWACP on completion of the study.

### Data collection

All study outcomes were measured at baseline, 12 weeks (intervention completion), and 24 weeks to assess program sustainability. The data were collected via online survey and virtual appointments were conducted by a trained research assistant (RA) blinded to group allocation. The online survey was sent using a unique link to the participant’s registered email address at weeks 0, 12, and 24. A virtual appointment between the RA and the participant occurred at baseline, week 12, and week 24 using Skype, Zoom, or Facetime where adverse event data were collected. Data on intervention compliance was collected by the cancer nurse specialist at weeks 6 and 12.

### Outcome measures for feasibility study

A detailed description of each outcome measure for feasibility has been published [18]. In summary, the feasibility outcomes measured included the proportions of women by socio-demographic characteristics (age, ethnicity, locality) who expressed interest but were not randomised; reasons for non-participation for those not randomised; the proportion of participants who discontinued the intervention; and reasons for intervention discontinuation. Participants were asked to fill out the questionnaire planned for the main study which included the Short Form-36 [31], Distress Thermometer [32], Female Sexual Function Index [33], Greene Climacteric Scale [34], Food Variety Checklist [35], Godin Leisure-Time Exercise Questionnaire [36], Pittsburgh Sleep Quality Index [37], and Body Image Scale [38]. Participants’ perceptions of the acceptability, accessibility, and likely future uptake of the intervention; measurement burden (time taken to complete questionnaires); and general study processes were included in the questionnaire. The data on efficacy outcomes from the questionnaires will be analysed in an individual patient meta-analysis in a subsequent paper, but the completeness of the efficacy data is reported here. We also collected self-reported information on access to support services outside of the study by the control group. We explored the timeframes for the delivery of the trial by assessing the time from submission of participant expression of interest to consent; time from registration to randomisation; time from completion of baseline assessment to randomisation; and time from randomisation to intervention start.

### Quality assurance and ethical compliance

This study was approved by the Southern Health and Disability Ethics Committee (Reference: 19/STH/215). The intervention cancer nurse specialist received a self-directed protocol manual. Case review by the project manager of at least one session per month monitored adherence to study protocols, with the nurse specialist completing a checklist at the end of each session to indicate the strategies used. All study data were entered by either the participant (questionnaires) or the CTNZ RA via the ZEDOC electronic Participant Reported Outcome Measures (ePROM platform) provided by The Clinician. The study adhered to Good Clinical Practice guidelines, including standard operating procedures for measurements, intervention delivery, and data collection. The RA received data collection training and was also audited monthly. Participants who withdrew from the study were encouraged to provide follow-up data. Information on reasons for withdrawal was collected.

### Data analysis

Quantitative data were analysed descriptively using proportions with 95% confidence intervals. Qualitative (free-text survey) data collected at weeks 12 and 24 were content analysed to determine measurement burden, acceptability, potential translation, and ease of intervention access.

### Sample size justification

Most outcome measures for this feasibility study are proportions calculated across both treatment and control groups. A sample size of 60 participants provides estimates with a margin of error (half the width of a 95% confidence interval) of 0.13. For proportions measured only on the intervention group, the margin of error with 30 participants is 0.19.

## Results

### Accessibility, acceptability, uptake, representativeness, and success of recruitment strategy

The Kōwhai Study was advertised between July 2020 and February 2021 through presentations to cancer support organisations (Cancer Society Auckland and Northland, Pinc and Steel, Breast Cancer Foundation NZ); breast cancer treatment services in Auckland and Hamilton; Facebook posts (Pinc and Steel and Breast Cancer Foundation NZ); and the Breast Cancer Aotearoa Coalition e-newsletter.

Recruitment was delayed 5 months due to COVID-19 but the target of 60 participants was recruited in 6 months (August 2020 to February 2021). Of 97 potential participants, 79 (81%) registered via the study website and the remainder contacted the study RA. Fifteen could not be reached and 12 were ineligible (receiving cancer treatment (*N* = 5), within the 4-week treatment completion window (*N* = 5), about to start radiation therapy (*N* = 1), metastatic disease (*N* = 1)). One enrolled in the Kōwhai Māori sub-study (*reported separately*), seven declined, and two withdrew after consent but before randomisation. Hence, 60 of 97 screened (62%) were successfully recruited and randomised.

Of the 60 participants who consented, the median time from submission of expression of interest to informed consent was 10 days; for 13 participants, the delay was more than 3 weeks. The median time from consent to randomisation was 11 days; for 11 (35%) participants, the delay between consent and randomisation was over 2 weeks. The median time from completion of baseline questionnaire to randomisation was 6.5 days. Five participants experienced a delay of over 2 weeks; hence, their randomisation took place shortly after the virtual appointment with the RA. Further reasons for delays were not recorded in the database.

Limited data on age, ethnicity, and locality were available for the women who registered their interest but were not consented. Participants were recruited nationally from 15 of the 20 District Health Board regions. Māori in Auckland were offered enrolment in the ‘Māreikura Tū Kōwhai Māori’ sub-study leading to fewer Māori participants in this feasibility study (Table 1). There was an imbalance in ethnicity between intervention and control groups, which must have occurred by chance given concealed allocation. Participants represented a range of education, income, and area-level deprivation, although those in the lowest categories of education and income, and the highest level of deprivation, were underrepresented in both groups.

**Table 1:** Comparison of demographic characteristics of participants and non-participants

	Treatment allocation			
	Wellness program (***N*** = 30)		Standard of care (*N* = 30)	
	*N*	Percent	*N*	Percent
**Age group (years)**				
25–34	6	(20.0)	2	(6.7)
35–44	13	(43.3)	14	(46.7)
45–54	11	(36.7)	14	(46.7)
**Prioritised ethnicity**				
European	26	(86.7)	19	(63.3)
Māori	0		2	(6.7)
Pacific	0		3	(10.0)
Other	4	(13.3)	6	(20.0)
**Marital status**				
Single	5	(16.7)	4	(13.3)
Married/de facto	23	(76.7)	23	(76.7)
Divorced	2	(6.7)	3	(10.0)
**Employment status**				
Full-time paid employee	13	(43.3)	12	(40.0)
Part-time paid employee	2	(6.7)	9	(30.0)
Unemployed	7	(23.3)	7	(23.3)
Other	8	(26.7)	2	(6.7)
**Highest qualification**				
Secondary school (NCEA level 1 to 3)	4	(13.3)	4	(13.3)
Trade/Technical certificate or diploma	8	(26.7)	4	(13.3)
Bachelor degree	8	(26.7)	10	(33.3)
Post-graduate degree	10	(33.3)	12	(40.0)
**Annual household income (per year)**				
< $36,500	3	(10.0)	4	(13.3)
$36,500 to $64,399	6	(20.0)	9	(30.0)
$64,400 to $97,599	6	(20.0)	6	(20.0)
$97,600 to $142,799	7	(23.3)	3	(10.0)
> $142,800	8	(26.7)	8	(26.7)
**Index of multiple deprivation (quintile)**				
1 (least deprived)	8	(26.7)	9	(30.0)
2	6	(20.0)	5	(16.7)
3	2	(6.7)	6	(20.0)
4	11	(36.7)	7	(23.3)
5 (most deprived)	3	(10.0)	3	(10.0)

The clinical characteristics of the participants are shown in Table 2. The majority had undergone mastectomy, chemotherapy, and localised radiotherapy, and were on endocrine therapy (most commonly tamoxifen and/or goserelin). Over 80% self-reported anxiety at baseline.

**Table 2:** Medical history and previous cancer treatment

	Treatment allocation				
	Wellness program (***N*** = 30)		Standard of care (*N* = 30)		
	*N*	Percent	*N*		Percent
**Time since diagnosis (years)**					
0–2	21	(70.0)	19		(63.3)
3–5	6	(20.0)	8		(26.7)
> 5	2	(6.7)	2		(6.7)
Unknown	1	(3.3)	1		(3.3)
**Type of surgical treatment**					
Mastectomy, unilateral	15	(50.0)	14		(46.7)
Mastectomy, bilateral	6	(20.0)	3		(10.0)
Breast-conserving surgery	9	(30.0)	13		(43.3)
**Chemotherapy**					
No	8	(26.7)	6		(20.0)
Yes	22	(73.3)	24		(80.0)
**Radiotherapy (localised)**					
Yes	24	(80.0)	25		(83.3)
No	6	(20.0)	5		(16.7)
**Targeted therapy (Herceptin)**					
Yes	5	(16.7)	8		(26.7)
No	25	(83.3)	22		(73.3)
**Endocrine therapy**					
Yes	21	(70.0)	21		(70.0)
No	9	(30.0)	9		(30.0)
Baseline medical conditions*					
Headaches/migraines	7	(23.3)	5		(16.7)
Stress incontinence	2	(6.7)	5		(16.7)
Back problem	6	(20.0)	7		(23.3)
Heart disease	2	(6.7)	3		(10.0)
Irritable bowel problem	7	(23.3)	4		(13.3)
Thyroid disorder	0	(0.0)	5		(16.7)
Arthritis or rheumatism	2	(6.7)	3		(10.0)
Bone or joint problem	4	(13.3)	3		(10.0)
Clinical depression	6	(20.0)	8		(26.7)
Anxiety disorder	24	(80.0)	25		(83.3)

*More than one condition could be selected

### Sustainability and adherence of intervention over time

Of the 30 participants in the intervention group, one withdrew before their first consultation, and one withdrew after the first consultation citing ‘too many other commitments’. The remaining 28 completed all the intervention consultations. The intervention started within seven days of randomisation for 14 participants. For 11 participants, commencement occurred within 2 weeks, but for three, the start was delayed by over 2 weeks. Week 6 and week 12 consultations were also behind schedule for some. Whilst the protocol allowed a 6-day intervention start window and expected completion by 90 days, all but two exceeded this with a maximum of 140 days. The longest delays were due to unexpected surgeries (*N* = 2), participant rescheduling (*N* = 1), and unknown reasons (*N* = 2).

### Measurement burden

**Fig. 1:**
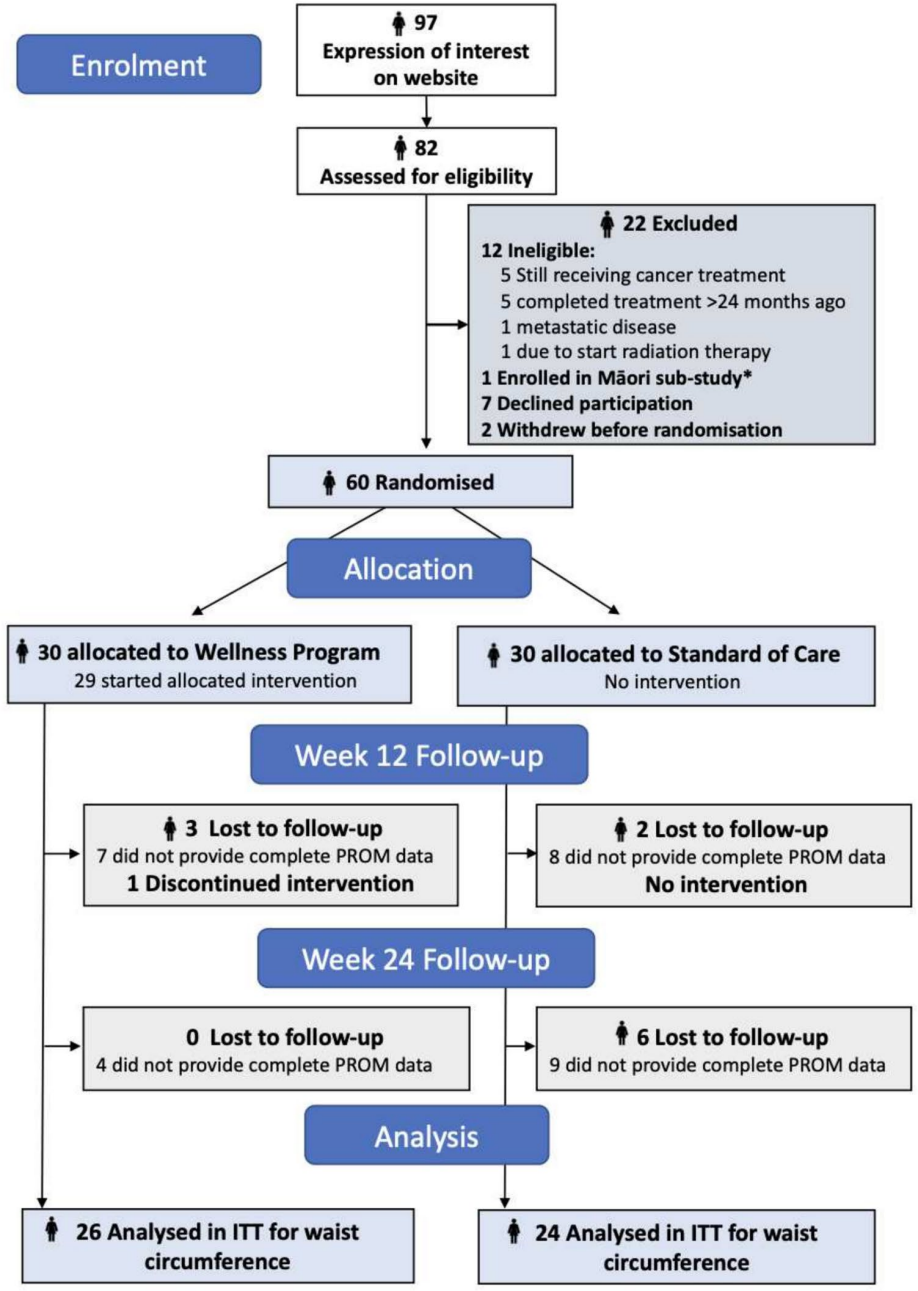
Consort flow diagram.*The Kōwhai Māori sub-study ‘Māreikura Tū Kōwhai Māori’ was completed alongside the main RCT feasibility study and is reported separately

Retention data is shown in the CONSORT flowchart (Fig. 1). All baseline questionnaires were completed. At week 12, complete data was available for 23 (76.7%) intervention and 22 (73.3%) control participants, at week 24 for 26 (86.7%) and 21 (70%) respectively. A database error prevented the Female Sexual Function Index being presented to participants at follow-up, so this was not completed after baseline (Table 3). Participants provided free-text feedback on questionnaire completion time and difficulties. Most took 15–20 min at each timepoint (range 10–45 min), with all but three participants reporting no issues. Those that did noted difficulties with categorical or Likert scale responses for complex items.

**Table 3:** Completeness of follow-up data

	Baseline		Week 12		Week 24	
	Wellness program (*N* = 30)	Standard of care (*N* = 30)	Wellness program (*N* = 30)	Standard of care (*N* = 30)	Wellness program (*N* = 30)	Standard of care (*N* = 30)
Demographics and medical history	30	30	26	24	27	21
Short Form-36 (SF36) [31]	30	30	25	23	26	21
Distress Thermometer (DT) [32]	30	30	25	22	26	21
Female Sexual Function Index (FSFI) [33]	30	30	0	0	0	0
Greene Climacteric Scale (GCS) [34]	30	30	23	22	26	21
Food Variety Checklist (FVC) [35]	30	30	23	22	26	21
Godin Leisure-Time Exercise Questionnaire (GLTEQ) [36]	30	30	23	22	26	21
Pittsburgh Sleep Quality Index (PSQI) [37]	30	30	23	22	26	21
Body Image Scale (BIS) [38]	30	30	23	22	26	21
Feasibility questionnaire	NA	NA	23	22	26	21
Intervention feasibility questionnaire	NA	NA	NA	NA	21	NA
Control feasibility questionnaire	NA	NA	NA	NA	NA	17

### Prevalence of components of intervention in control group

In both study arms, 25 (83% per arm) participants reported using either zero or one support service at baseline (Supplementary Table 4). During follow-up, use of support services outside the intervention was greater for those in the intervention group than control group; therefore, there was no indication of contamination of the intervention effect due to cross-over.

### Feasibility and acceptability of study method and intervention

Most participants reported no issues with the way the study was undertaken, with five control group participants noting how smoothly the study ran from their perspective. Whilst one intervention participant reported digital access issues, three participants indicated that the combination of an online version of the intervention that enabled ready reference back to the hard copy of the program, that was also provided, was helpful and convenient. All participants in the intervention group indicated in the free-text responses that the intervention was feasible and a valuable aid to recovery after cancer treatment, although one participant commented that the physical activity modules were ‘too involved’, and three others perceived them as ‘too basic’. Five intervention participants signalled that the three individualised Zoom coaching sessions with a cancer professional were particularly welcomed, and that the flexibility of coaching content and timing was a particular highlight. One participant in the intervention group perceived a 12-week intervention as ‘too long’ but did not indicate an alternative timeframe.

## Discussion

The Kōwhai Study’s feasibility assessment of the YWWACP in Aotearoa NZ provided key insights into accessibility, acceptability, and uptake including recruitment challenges, cultural adaptation, and measurement burden. These findings will inform the design of a future larger RCT. Whilst the study affirmed intervention acceptability, it also highlighted systemic barriers to recruiting diverse populations. It emphasises the need for culturally grounded co-design and streamlined data collection to improve equity and scalability. This discussion integrates feasibility findings with broader cancer survivorship literature, identifying implications for future research, and the study’s role in addressing health inequities amongst younger breast cancer survivors.

Effective participant recruitment was crucial in assessing the feasibility of the study. The Kōwhai study successfully engaged participants through partnerships with established cancer organisations and healthcare providers. Whilst this approach efficiently achieved the target sample size of *N* = 60 in 6 months (even during COVID-19 disruptions), as in the Australian studies, we failed to reach challenged populations such as Pacific Islander, Asian, and socioeconomically disadvantaged groups [39]. We acknowledge that Māori and Pacific Islander women experience specific demographic and cultural challenges in terms of cancer determinants, access to treatments, cancer outcomes, and cancer aftercare approaches. To address this, the YWWACP was redesigned and culturally adapted by Māori women as the ‘Mareikura, Women’s Wellness after Cancer Program’, with the aim of piloting it to assess acceptability, uptake sustainability, adherence, and potential for a national roll-out. Kaupapa Māori research methods informed the study design and qualitative analysis [39].

The intervention, combining health professional consultations with the YWWACP journal and website, demonstrated high acceptability. This was reflected in strong participant retention and satisfaction. As quoted by one participant, ‘Absolutely nothing prepared me for the mental hell that is life after treatment and 2019 felt worse than 2018. Had this programme been available [in Aotearoa NZ], it would have been an amazing tool for me during that horrible year’. A key to the program’s success appears to be its flexibility and choice of delivery medium, enabling women to choose when and where they undertook intervention activities [40]. Online tailored, flexible delivery is important for successful lifestyle interventions in clinical populations [41–43]. Some women expressed a desire for peer connection within the intervention to ease the isolation from their usual social circles due to their cancer diagnosis. Healthcare professional-facilitated structured peer connection can significantly empower breast cancer survivors, with ‘exchanging information’ and ‘finding recognition’ being key predictors of positive outcomes [44]. This aligns with meta-ethnography findings [45] that peer support creates therapeutic connections but requires professional oversight to manage emotional risks and provision of non-evidence based, anecdotal and potentially unsafe information.

The feasibility study aimed to reduce measurement burden for participants by revising the instrument battery based on participant feedback from previous WWACP trials. Completion rates were high at baseline (100% for both groups) and remained strong at week 12 (76.7% intervention/73.3% control) and week 24 (86.7% intervention/70.0% control). The lower week 24 completion rate in the control group is not unexpected but warrants further exploration, and consideration of how to increase control group survey completion to avoid bias in the future. Participants reported manageable questionnaire completion times (15–20 min on average, range 10–45 min) and indicated minimal difficulty with qualitative data, documenting comprehensive feedback about the intervention and intervention tools. Completion rates align with existing research on measurement burden in clinical trials, where shorter questionnaires and electronic data collection improve completion rates [46]. Whilst the Kōwhai study prioritised brevity, qualitative feedback suggested some survey items remained complex. For example, participants struggled with distinguishing types of treatment and surgical procedures. Dropdown boxes would have proved useful here and will be utilised in future to ensure accurate data collection. The answers provided by several women to the Godin Leisure-Time Exercise Questionnaire [36] suggested they had misinterpreted the question. Further exploration of the validation of the questionnaire for Aotearoa NZ is required.

Strengths of the Kōwhai Study include its focus on the feasibility and cultural adaptation of delivering the YWWACP, as well as providing insights into recruitment, retention, and intervention implementation in this population. The study employed a randomised design, enhancing the rigor of feasibility assessment. The limitations of this study include it not being powered to detect significant intervention effects, as the primary aim was feasibility. The delays in randomisation highlight potential logistical challenges and the reasons for these delays were not systematically recorded, limiting the ability to fully understand and mitigate them in future studies. Participant self-selection may have also introduced bias, as those most interested in lifestyle interventions may have been more likely to enrol. Finally, some participants reported that physical activity online modules were too involved or too basic, as the modules were not individually tailored.

## Conclusion

Feasibility testing of the YWWACP in Aotearoa NZ was successful. The intervention was highly valued by young women treated for breast cancer. The study processes are feasible, as demonstrated by timely recruitment, good acceptability, and accessibility. A country-wide study based on this feasibility work is planned for Aotearoa NZ**.**

## Supplementary Information

Below is the link to the electronic supplementary material.

Supplementary file1 (DOCX 42 KB)

## Data Availability

No datasets were generated or analysed during the current study.
