# Supplementary material for: The younger women’s wellness after cancer program: results from feasibility testing in Aotearoa New Zealand (the ‘Kōwhai study’)
Source: Support Care Cancer. 2025 Jun 5;33(7):546. doi: 10.1007/s00520-025-09601-8 (PMC12141147; doi:10.1007/s00520-025-09601-8)
Supplement: Supplementary file 1 — Supplementary file1 (DOCX 42 KB) [file 520_2025_9601_MOESM1_ESM.docx]

**Title**

The Younger Women’s Wellness After Cancer Program: Results from Feasibility Testing in Aotearoa New Zealand (the ‘Kōwhai Study’)

**Journal Name**

Supportive Care in Cancer

**Author Names and Affiliations**

Janine P. PORTER-STEELE - Griffith Health Group Executive, Gold Coast campus, Griffith University, Australia; School of Nursing, Midwifery and Social Work, University of Queensland, Australia; Wesley Choices Cancer Support Centre, The Wesley Hospital, Australia

Katrina J. SHARPLES - Cancer Trials New Zealand, The University of Auckland, Aotearoa New Zealand; University of Otago, Aotearoa New Zealand

Bobbi B. LAING - Cancer Trials New Zealand, The University of Auckland, Aotearoa New Zealand; School of Nursing, University of Auckland, Aotearoa New Zealand

Sarah BENGE - Cancer Trials New Zealand, The University of Auckland, Aotearoa New Zealand

Sarah M. BALAAM - Griffith Health Group Executive, Gold Coast campus, Griffith University, Australia; School of Nursing, Midwifery and Social Work, University of Queensland, Australia

Natalie K. VEAR - School of Nursing, Midwifery and Social Work, University of Queensland, Australia

Michael P.N. FINDLAY - Cancer Trials New Zealand, The University of Auckland, Aotearoa New Zealand

Ian D. CAMPBELL - Department of Surgery, Waikato Hospital, Aotearoa New Zealand

Marion J.J. KUPER-HOMMEL - Department of Oncology, Waikato Hospital, Aotearoa New Zealand

Debra J. ANDERSON - University of Technology Sydney, Australia

David J. PORTER - Department of Oncology, Auckland Hospital, Aotearoa New Zealand

Alexandra L. MCCARTHY - Griffith Health Group Executive, Gold Coast campus, Griffith University, Australia; School of Nursing, Midwifery and Social Work, University of Queensland, Australia; Mater Research Institute, Australia; Wesley Research Institute, The Wesley Hospital, Australia

**Corresponding Author E-mail Address**

[n.vear@uq.edu.au](mailto:n.vear@uq.edu.au)

**Supplementary Table 1.** Core targeted health knowledge and behaviours

| **Behaviour** | **Recommendations** | **Rationale** |
| --- | --- | --- |
| Physical activity | Be moderately physically active, equivalent to brisk walking, for ≥ 30 minutes daily. As fitness improves, aim for ≥ 60 minutes of moderate (or for ≥ 30 minutes of vigorous)  physical activity every day. | Physical activity of longer duration or greater intensity is more beneficial. All forms of physical activity protect against some cancers, as well as against weight gain, overweight, and obesity. |
| Diet | Eat mostly foods of plant origin. Limit consumption of energy dense foods. Avoid sugary drinks. Limit intake of red meat and avoid processed meat. | The evidence indicates that most diets that are protective against cancer mainly comprise foods of plant origin. Energy-dense foods and sugary drinks contribute to obesity. The evidence also indicates foods  of animal origin are nourishing and healthy if consumed in modest amounts. |
| Alcohol | If alcoholic drinks are consumed, limit consumption to no more than one drink per day. | The evidence on balance justifies alcohol abstinence, although some evidence indicates that modest amounts of alcohol could reduce the risk of coronary heart disease. |
| Body fatness | Be as lean as possible within the normal weight range. Avoid weight gain and  increases in waist circumference. | Maintenance of a healthy weight could be one of the most important ways to protect against treatment-  related chronic diseases. |

**Supplementary Table 2.** YWWACP intervention content and delivery strategies

| **Week/s** | **Delivery Strategies** | **Content** |
| --- | --- | --- |
| 1 | Individual or group virtual consultation delivered by cancer nurse as preferred by participants | Virtual consultation delivered by specialist cancer nurse: Phone coaching, iBook, health education material, website, and email. Introduction to website and iBook. Development of tailored health education based on agreement between nurse and participant on an individualized plan and goals. Discussed healthy weight measures and associated risk factors i.e., BMI, waist/hip ratio. Discussed menopause, stress,  sleep and other concerns and appropriate screening. |
| 3,9 | Email/phone | At Weeks 3 and 9 follow up email sent from the consultation nurse and phone call if requested to the participant. This phase of communication enquired how the  participant was progressing with the program, reviewed their individualised plan, identified barriers and plans for completion of the program. |
| 6 | Virtual consultation delivered by nurse | Through health education and motivational interviewing, this virtual appointment addressed:   - Review of plan and goals - Behavioural relapse prevention strategies - Answer questions.   The goals that the woman set in the first consultation were reviewed and revised as necessary, including discussion of a personal action plan and identification of barriers. Issues or concerns raised by the participant addressed by nurse. |
| 12 | Virtual consultation delivered by nurse | Discussion and review of how the participant found the program and whether individual goals were met including biophysical measurements. The participant encouraged to keep up any positive behaviour change following the formal program. Women often set further personal goals to self-monitor. Relapse prevention included, with encouragement of importance of maintaining positive behaviour change following formal completion of the program. This final appointment also addressed:   - Review of plan and goals and set future goals. - Discussion of biophysical measurements. |

**Supplementary Table 3.** Effectiveness outcome measures, instruments, modes, and times of administration

| Outcome measure | Variable, previous instrument, new instrument, item numbers | | Time Point | | | Administration mode | |
| --- | --- | --- | --- | --- | --- | --- | --- |
|  |  |  | T_0_ | T_1_ | T_2_ | Online Survey | RA |
| Socio-demographics | Age, marital status, employment status, income, ethnicity, medical history | | X |  |  | X |  |
| Height (once only), weight, waist circumference | World Health Organisation standardised protocols collected virtually under supervision of the RA | | X | X | X |  | X |
|  | **Previous measures**  **(340 items)** | **New measures**  **(125 items)** |  | | | | |
| Health-related QoL | FACT-G and SF36 (63 items) | SF36 (36 items) | X | X | X | X |  |
| Depression | CES-D (20 items) | DT (1 item) distress incidence and impact | X | X | X | X |  |
| Anxiety | Zung SAS (20 items) |  | X | X | X | X |  |
| Sexual function | FSFI-BC (19 items) | FSFI (19 items) | X | X | X | X |  |
| Menopausal symptoms | GCS (22 items) | GCS (22 items) | X | X | X | X |  |
| Diet | FFQ (103 items) | FVC (14 items) | X | X | X | X |  |
| Physical Activity | IPAQ (27 items) | GLTEQ (4 items) | X | X | X | X |  |
| Sleep | PSQI (19 items) | PSQI (19 items) | X | X | X | X |  |
| Cognitive function | FACT-Cog (37 items) | Not measured |  |  |  |  |  |
| Body image | BIS (10 items) | BIS (10 items) | X | X | X | X |  |
| Free-text survey | Nil | Perceptions of measurement burden, intervention effectiveness |  | X | X | X |  |
| T_0_ – Baseline; T_1_ – 12 weeks; T_2_ – 24 weeks  BIS – Body Image Scale [38]; CES-D – Center for Epidemiologic Studies - Depression Scale; DT – Distress Thermometer [32]; FACT-G – Functional Assessment of Cancer Therapy - General; FFQ – Food Frequency Questionnaire; FSFI – Female Sexual Function Index [33]; FVC – Food Variety Checklist [35]; GCS – Greene Climacteric Scale [34]; GLTEQ – Godin Leisure-Time Exercise Questionnaire [36]; IPAQ – International Physical Activity Questionnaire; PSQI – Pittsburgh Sleep Quality Index [37]; QoL – quality of life; RA – research assistant; SF36 – Short Form-36 [31] ; Zung SAS - Zung Self-rating Anxiety Scale | | | | | | | |

### Supplementary Table 4. Use of support services – number of support services used during follow-up

| **Number of support services** | **Baseline** | | | | **Week 12** | | | | **Week 24** | | | |
| --- | --- | --- | --- | --- | --- | --- | --- | --- | --- | --- | --- | --- |
|  | **Wellness Program (N=30)** | | **Standard of Care (N=30)** | | **Wellness Program (N=30)** | | **Standard of Care (N=30)** | | **Wellness Program (N=30)** | | **Standard of Care (N=30)** | |
|  | **N** | **(%)** | **N** | **(%)** | **N** | **(%)** | **N** | **(%)** | **N** | **(%)** | **N** | **(%)** |
| 0 | 15 | (50.0) | 14 | (46.7) | 12 | (40.0) | 13 | (43.3) | 14 | (46.7) | 14 | (46.7) |
| 1 | 10 | (33.3) | 11 | (36.7) | 8 | (26.7) | 7 | (23.3) | 6 | (20.0) | 5 | (16.7) |
| 2 | 3 | (10.0) | 3 | (10.0) | 4 | (13.3) | 3 | (10.0) | 5 | (16.7) | 1 | (3.3) |
| 3 | 1 | (3.3) | 2 | (6.6) | 1 | (3.3) | 0 | (0.0) | 1 | (3.3) | 1 | (3.3) |
| 4 | 1 | (3.3) | 0 | (0.0) | 1 | (3.3) | 0 | (0.0) | 0 | (0.0) | 0 | (0.0) |
| Unknown | 0 | (0.0) | 0 | (0.0) | 4 | (13.3) | 7 | (23.3) | 4 | (13.3) | 9 | (30.0) |
